# Impact of the Use of 2-Phospho-L Ascorbic Acid in the Production of Engineered Stromal Tissue for Regenerative Medicine

**DOI:** 10.3390/cells14141123

**Published:** 2025-07-21

**Authors:** David Brownell, Laurence Carignan, Reza Alavi, Christophe Caneparo, Maxime Labroy, Todd Galbraith, Stéphane Chabaud, François Berthod, Laure Gibot, François Bordeleau, Stéphane Bolduc

**Affiliations:** 1 Regenerative Medicine Division, Centre de Recherche du CHU de Québec-Université Laval, Québec, QC G1J 5B3, Canada; david.brownell@crchudequebec.ulaval.ca (D.B.); christophe.caneparo.1@ulaval.ca (C.C.); todd.galbraith@crchudequebec.ulaval.ca (T.G.); stephane.chabaud@crchudequebec.ulaval.ca (S.C.); francois.berthod@fmed.ulaval.ca (F.B.); 2 Centre de Recherche en Organogénèse Expérimentale de L’Université Laval/LOEX, Université Laval, Québec, QC G1J 5B3, Canada; laurence.carignan@crchudequebec.ulaval.ca (L.C.); reza.alavi@crchudequebec.ulaval.ca (R.A.); maxime.labroy.1@crchudequebec.ulaval.ca (M.L.); 3 Centre de Recherche sur le Cancer, Université Laval, Québec, QC G1J 5B3, Canada; 4 Oncology Division, Centre de Recherche du CHU de Québec-Université Laval, Québec, QC G1J 5B3, Canada; 5 Department of Surgery, Université Laval, Québec, QC G1V 0A6, Canada; 6 Université de Toulouse, CNRS UMR 5623, Laboratoire Softmat, 31062 Toulouse, France; laure.gibot@cnrs.fr; 7 Department of Molecular Biology, Medical Biochemistry and Pathology, Faculty of Medicine, Université Laval, Québec, QC G1V 0A6, Canada

**Keywords:** tissue engineering, extracellular matrix, sodium L-ascorbate, 2-phospo-L-ascorbate, human primary fibroblasts

## Abstract

Tissue engineering enables autologous reconstruction of human tissues, addressing limitations in tissue availability and immune compatibility. Several tissue engineering techniques, such as self-assembly, rely on or benefit from extracellular matrix (ECM) secretion by fibroblasts to produce biomimetic scaffolds. Models have been developed for use in humans, such as skin and corneas. Ascorbic acid (vitamin C, AA) is essential for collagen biosynthesis. However, AA is chemically unstable in culture, with a half-life of 24 h, requiring freshly prepared AA with each change of medium. This study aims to demonstrate the functional equivalence of 2-phospho-L-ascorbate (2PAA), a stable form of AA, for tissue reconstruction. Dermal, vaginal, and bladder stroma were reconstructed by self-assembly using tissue-specific protocols. The tissues were cultured in a medium supplemented with either freshly prepared or frozen AA, or with 2PAA. Biochemical analyses were performed on the tissues to evaluate cell density and tissue composition, including collagen secretion and deposition. Histology and quantitative polarized light microscopy were used to evaluate tissue architecture, and mechanical evaluation was performed both by tensiometry and atomic force microscopy (AFM) to evaluate its macroscopic and cell-scale mechanical properties. The tissues produced by the three ascorbate conditions had similar collagen deposition, architecture, and mechanical properties in each organ-specific stroma. Mechanical characterization revealed tissue-specific differences, with tensile modulus values ranging from 1–5 MPa and AFM-derived apparent stiffness in the 1–2 kPa range, reflecting the nonlinear and scale-dependent behavior of the engineered stroma. The results demonstrate the possibility of substituting AA with 2PAA for tissue engineering. This protocol could significantly reduce the costs associated with tissue production by reducing preparation time and use of materials. This is a crucial factor for any scale-up activity.

## 1. Introduction

Life expectancy is influenced by access to high-quality healthcare [1], underlining the importance of technological advancements in this field. In developed countries, people’s lifestyles have undergone a shift towards greater sedentariness, leading to a significant rise in chronic illnesses [2], potentially requiring more organ transplants. Additionally, life expectancy has increased with advancing technology, further increasing transplant organ demand [3]. Stringent regulatory standards have resulted in a greater demand for organ grafts, surpassing the available supply [4]. In light of these pressures on healthcare systems, alternative strategies must be explored.

One such strategy is tissue engineering, which emerged as a groundbreaking concept 30 years ago in response to the undersupply of donor organs [5]. This approach enables the in vitro reconstruction of organs for transplantation into patients. Despite three decades of development, few products have become available to the public, often due to stringent regulation and difficulties related to scale-up. Thus, protocol optimization and standardization are needed to improve clinical translation.

Over the past three decades, the field has advanced considerably. Tissue engineering has seen remarkable progress, evolving reconstructed tissues from mainly inert biomaterials to structures incorporating host cells and surface functionalization, approaching the properties of native tissues [6,7]. Three-dimensional models have shown significant promise in producing substitute organs, as well as preclinical models. However, numerous challenges remain, particularly concerning the scale-up of laboratory techniques [6]. Currently, mostly cell-free constructs have been approved by regulatory bodies. Indeed, 86% of cell-based tissue engineering products have failed to pass the U.S. Food and Drug Administration (FDA) approval [8], and currently only eight products are FDA-approved [9,10,11,12,13,14,15]. Six of the eight approved products are used to treat skin wounds [9,11,12,13,15]. This is in part due to inexistant infrastructure for approving tissue-based products, which has only recently been pioneered by major regulatory bodies such as the FDA [16]. Furthermore, tissue-based products are mostly developed in academia, where regulatory expertise is lacking. Standardization of protocols could help academics to increase chances of passing the reglementary barriers.

A key factor influencing tissue-engineered construct quality is the production of the extracellular matrix (ECM), particularly collagen, by stromal cells. Ascorbic acid (AA), or vitamin C, is a critical building block of collagen, and its deficiency is associated with impaired ECM deposition and collagen fibrillogenesis [17]. AA is a cofactor of prolyl and lysyl hydroxylases, which are essential in the post-transcriptional maturation of collagen [18]. Thus, AA is often added in the form of sodium ascorbate to growth media in tissue engineering applications to stimulate the production and maturation of an endogenous ECM by fibroblasts [19]. This applies to tissues of various organ sources such as skin [20], bladder [21], and vagina [22], to name a few. Other mesenchymal cells may also produce endogenous ECM under AA stimulation. For instance, cartilage tissue engineering may use AA to stimulate matrix production by primary chondrocytes [23]. A cartilage-like ECM is deposited by chondrocytes containing collagens II and VI, and glycosaminoglycans (GAGs) [24]. Adipose-derived stem/stromal cells (ASCs) have also been shown to deposit collagen to form ECM sheets when stimulated with AA, showing promise as a bioactive material thanks to their regenerative and immunomodulatory secretome [25]. Other mesenchymal cells, such as keratocytes from the cornea [26], gingival [27], bronchi, [28] or ligament fibroblasts [29], have been shown to secrete and deposit collagen-rich ECM in the presence of AA.

In particular, fibroblast-driven ECM deposition is pivotal to the self-assembly method. The use of AA is paramount to stimulate fibroblasts to produce ECM in vitro. The ECM formed is organ-specific, thus advantageous for tissue-specific reconstruction. Epithelial cells seeded on cell-produced ECM have been shown to differentiate to recapitulate native histology [30,31,32]. This ECM deposition is all the more crucial when it is deposited by cells without the support of a scaffold, as in the case of the self-assembly method, which relies entirely on the ability of cells to produce a tissue without the use of exogenous materials [33]. Based on this method, some models have elaborated protocols to incorporate various other cell types such as immune cells and endothelial cells to mimic the native microenvironment [34]. The self-assembly method has also been used in the creation of “human textiles”, where the cell-produced ECM from skin fibroblasts has been devitalized and spun to form ECM yarn that can be woven on a loom to form various constructs, from biological suture material to mesh implants [35,36,37].

Despite its essential role, AA presents technical challenges due to its instability. AA is unstable in aqueous solution, with a half-life that depends on the specific conditions [38]. The half-life of AA has been shown, for example, to be 1 day in culture conditions or 7 days at 5 °C [39], meaning that fresh solutions must be made at each medium change, 3 times per week to control media concentration. This poses a challenge for the production of engineered tissues, as it increases both the cost and time spent at the bench. Indeed, each solution preparation requires time and materials to weigh, dissolve, and sterile-filter before use. A more stable form of AA, 2-phospho-L-ascorbate (2PAA), containing a phosphate group in the molecule, has been shown to only marginally degrade after 3 days in culture or 1 month at 5 °C [39]. 2PAA allows the formation of connective tissue-like structures in fibroblast cultures [39], but it remains unclear if 2PAA constitutes an adequate substitute for AA in tissue engineering.

To investigate this question, in this study, we show that 2PAA can be used to produce engineered tissues that are equivalent to those produced with AA. Since the amount of ECM deposited by cells, and the quality of its organization are essential parameters to produce strong and functional tissues, the physico-chemical properties of AA and 2PAA solutions were compared to evaluate molecule stability in culture and storage conditions. The impacts of using 2PAA for ECM production by fibroblasts was then evaluated by producing dermal, bladder, and vaginal tissues using the self-assembly technique. The biochemical properties and the histoarchitecture of the tissues were then evaluated.

## 2. Materials and Methods

### 2.1. Ethics Statement

All procedures involving patients were conducted according to the Declaration of Helsinki and were approved by the Research Ethics Committee of CHU de Québec-Université Laval (2012-1341). Donors’ consent for tissue harvesting was obtained for each specimen (DR-002-1190). Experimental procedures were performed in compliance with the CHU de Québec guidelines.

### 2.2. Preparation of AA and 2PAA Stock Solutions

**Figure 1:**
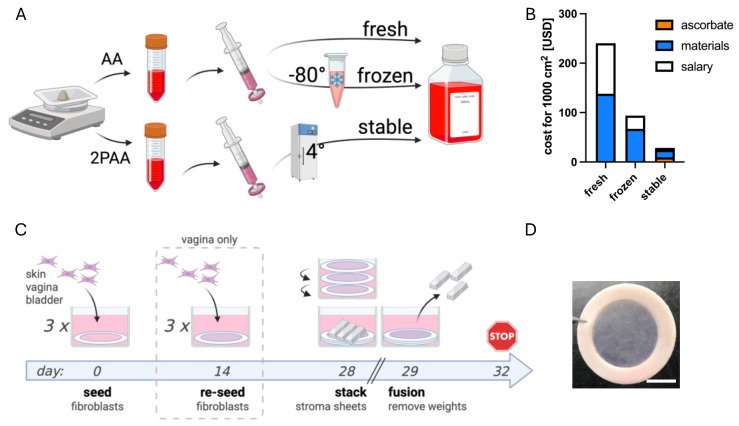
Production of tissue-engineered stroma with the self-assembly technique. Schematic description of AA and 2PAA solution preparation and storage (**A**). AA-related costs associated with tissue production using the self-assembly technique (**B**). Ascorbate represents molecule costs; materials represent disposables costs; the salary is based on labour time set at 35 CAD/hour. Schematic description of tissue culture protocol used in self-assembly tissue engineering (**C**). Example of macroscopic appearance of tissues produced (**D**). Scale bar represents 1 cm.

AA ((+)-sodium-L-ascorbate, Sigma-Aldrich, Saint Louis, MO, USA) and 2PAA (2-phospho-L-ascorbic acid trisodium salt, Sigma-Aldrich) stock solutions were prepared at a 200X working concentration, at 10 mg/mL in DMEM (Dulbecco’s Modified Eagle’s Medium, Corning, Corning, NY, USA) with or without phenol red. The solutions were sterilized with 0.22 µm filters and either used fresh or aliquoted and stored at −80 °C for AA or stored at 4 °C for 2PAA to imitate media storage in a refrigerator (Figure 1A).

### 2.3. Direct Spectrophotometric Absorbance Measurement of AA and 2PAA

AA and 2PAA stock solutions (10 mg/mL) were reconstituted in DMEM without phenol red and stored protected from light at 4 °C, −80 °C, or 37 °C in a humidified 5% CO_2_ incubator. At the moment of the measurement, the solutions were diluted 200-fold with the same medium, respectively, and kept at 4 °C, −80 °C, or 37 °C to a final concentration of 50 µg/mL, similar to that used for cell culture experiments. Then, 2.7 mL of these solutions were placed into 10 mm path length quartz cuvettes before being analyzed. Measurements were performed at 25 °C on a UV–vis scanning spectrophotometer (HP1 8452 single beam, Olis, Bogart, GA, USA) in the 230–820 nm wavelength range, with a slot of 2 nm, and an integration time of 0.2 s. For illustrative purposes, the raw data were normalized to a maximum absorption peak equal to 1.

### 2.4. Cell Culture

Dermal biopsies were taken from one healthy donor (female, 34 y/o) undergoing plastic surgery. Vaginal mucosa and bladder biopsies were obtained from patients undergoing surgery for benign pathologies (female 32 y/o and pre-pubertal male, respectively).

The specimens were rinsed abundantly in Phosphate Buffer Solution (PBS) containing 100 U/mL penicillin (Sigma-Aldrich), 25 mg/mL gentamicin (Schering, Pointe-Claire, QC, Canada), and 0.5 µg/mL Amphotericin B (Fisher Scientific, Ottawa, ON, Canada), cut into small strips and incubated overnight at 4 °C in a 500 mg/mL thermolysin solution (Sigma-Aldrich) diluted in a 4-(2-hydroxyethyl) 1piperazineethanesulfonic acid (HEPES; MP Biomedicals, Montreal, QC, Canada) buffer with 1 mM of CaCl2 pH 7.4 (Sigma-Aldrich) to digest the basal lamina. The epithelia were then manually separated from the stroma with tweezers. The stroma was incubated for 3 h in a 125 U/mL collagenase H solution (Boehringer Mannheim, Laval, QC, Canada) diluted in complete DMEM (cDMEM; DMEM supplemented with 10% fetal bovine serum (FBS; Avantor Seradigm FB Essence, Randor, PA, USA), 100 U/mL penicillin, and 25 mg/mL gentamicin) at 37 °C in a humidified 8% CO_2_ incubator.

Dermal, vaginal, and bladder fibroblasts (FD, FVa, and FB, respectively) were all grown in cDMEM in a humidified incubator at 37 °C with 8% CO_2_, with the media changed 3 times per week and passaged using trypsin-EDTA.

### 2.5. Quantification of Pro-Collagen 1α1 Secretion

FD were seeded in 24-well plates at near confluency (4 × 10^4^ cells/cm^2^) and cultured for 3 days in cDMEM. The media were then changed according to the experimental condition. For the evaluation of donor variation, 5 dermal fibroblast populations were used, and cDMEM was supplemented with 50 µg/mL AA. For the FBS concentration assay, 10, 15, 20, 25, and 30% FBS were added to DMEM with 100 U/mL penicillin, and 25 mg/mL gentamicin, and 50 µg/mL AA. AA dose-response evaluation was performed with 25, 50, 100, 200, and 300 µg/mL AA in cDMEM. For the ascorbate variation test, AA at 50 µg/mL was either prepared fresh or frozen and stored, or stable 2PAA was used at 50 µg/mL. After 3 days of culture, the supernatant was collected, centrifuged at 2000× *g*, and frozen at −80 °C until used in an Elisa Human pro-collagen 1α1 kit (Abcam, Cambridge, UK) according to manufacturer’s instructions. n = 3 for all groups, except FD4, FD5, and 30% FBS, where n = 2.

### 2.6. Reconstruction of Stroma by Self-Assembly

FD, FVa, and FB at passages 3 to 5 were seeded in six-well culture plates (Falcon, Corning, NY, USA) with a ring of filter paper made from a Whatman Qualitative Filter Paper: Grade 4 circles of filter paper were placed at the bottom of each well to facilitate the handling of stromal sheets and to limit contraction. For dermal stroma, FDs were seeded at a density of 5 × 10^4^ cells/cm^2^ [40]. For the vaginal stroma, FVs were seeded at a density of 1 × 10^5^ cells/cm^2^ [41]. For the bladder stroma, FBs were co-seeded with FDs (80% FB, 20% FD) [21] for a total density of 5 × 10^4^ cells/cm^2^.

Paper rings were weighed down by custom stainless-steel air–liquid support rings. Cells were cultured in cDMEM supplemented with 50 µg/mL AA (fresh or frozen) or 2PAA (refrigerated). The culture medium was changed three times per week with the respective AA or 2PAA stock being added each time.

After 14 days, vaginal stromal sheets were reseeded similarly to the initial seeding. After a total of 4 weeks of culture, fibroblasts had secreted enough collagen to form stromal sheets. In some experiments, 3 stromal sheets were stacked for each condition to form a thick construct. Merocel instrument wipes (BVI Medical, Waltham, MA, USA) were trimmed and placed on top of stacked stroma sheets, and 3 stainless steel lingots (0.6 g each) were placed on top to assist in stromal sheet fusion. The lingots and surgical sponges were removed after 24 h, and the stroma were left 3 more days in culture for further sheet fusion. Figure 1C illustrates the tissue engineering process. Six replicate tissues were constructed for each condition (n = 6) except for the vagina with fresh AA, where n = 5, and for the skin and vagina with frozen AA, where n = 4 and n = 5, respectively.

### 2.7. Quantification of Collagen Secretion

Equal volumes of supernatants were separated by electrophoresis on sodium dodecyl sulfate (SDS)-10% polyacrylamide gels. A 5% methanol 10 mM 3-(Cyclohexylamino)-1-propanesulfonic acid (CAPS, Sigma) at pH 11 buffer solution was used to transfer proteins onto a polyvinylidene difluoride (PVDF) membrane (Bio-Rad Laboratories Ltd. Montreal, QC, Canada). Proteins were revealed using a rabbit anti-human Col-I polyclonal antibody (Rockland Immunochemicals, Gilbertsville, PA, USA). SuperSignal^®^ West Dura extended chemiluminescent substrate (Pierce, Fisher Canada, Nepean, ON, Canada) was used and light signal was detected by Fusion Fx7 (Vilbert-Lourmat, Marne-La-Vallée, France). Technical triplicates were evaluated using supernatant from independent tissues.

### 2.8. Matrix Metalloproteinase (MMP) Activity Quantification

Tissues were reconstructed as described, and before the sheet stacking step, cell culture supernatants were harvested and stored at −80 °C. The total MMP activity was determined in cell culture supernatants using the SensoLyte™ 520 Generic MMP assay kit (Anaspec, San Jose, CA, USA) without the use of 4-aminophenylmercuric acetate (APMA). This kit can simultaneously detect the activity of MMP-1, 2, 7, 8, 9, 12, 13, and 14. APMA was not used, therefore inactive forms of MMP were not detected. The results reflect the overall active MMP and metalloproteinase inhibitor balance. Corning 96-well black plates with flat bottom and a Thermo Electron Varioskan 3001 Fluorometer Spectrometer were used. Four replicate tissues were constructed for each of the three conditions. Four independent measures were taken for each replicate.

### 2.9. Quantification of Total Protein and Collagen Content

Single ply stromal sheets that had not been stacked were used to avoid detection saturation. Stromal samples were collected with a 6 mm biopsy punch to normalize surface area, and a Sirius Red/Fast Green Collagen Staining kit (Chondrex, Woodinville, WA, USA) was used to quantify total collagen and total non-collagenous proteins within the tissues according to the manufacturer’s protocol. Samples were quantified by spectrometry using a SPECTRAmax Plus (Molecular Devices, San Jose, CA, USA) at 540 and 605 nm.

### 2.10. Total DNA Quantification

Quantification of DNA was performed as described previously [42]. A 6 mm biopsy punch was used on single-sheet reconstructed stroma to normalize surface area. Biopsies were homogenized in 200 µL PBS with 1.6 U/mL proteinase K (Bio Basic, Markham, ON, Canada) and the volume was adjusted to 210 µL with a solution of 10 mM Tris pH7.5, 10 mM EDTA by incubating overnight at 56 °C. The proteinase K was then inhibited by incubating at 75 °C for 20 min. Then, 63 µL of the tissue homogenate was incubated with 3 µL of 20 mg/mL RNase (Invitrogen, Waltham, WA, USA) for 2 h at 37 °C to degrade the RNA. The solution was diluted with 54 µL of 10 mM Tris pH 7.5, 1 mM EDTA. DNA was quantified using the Quant-iT PicoGreen dsDNA Assay Kit (Invitrogen) according to the manufacturer’s protocol. Technical triplicates were evaluated, using independent tissues.

### 2.11. Histological Analysis

All constructs were fixed in a 3.7% neutral buffered formaldehyde solution and embedded in paraffin. Histological sections, 5 mm thick, were sectioned and stained with Masson’s Trichrome. Samples were observed at 10X with a Zeiss Axio Imager M2 microscope equipped with an AxioCam HR Rev3 camera (Zeiss, Oberkochen, Germany). Images were processed with AxioVision 40 V4.8.2.0 software (Zeiss), and scale bars were added with ImageJ 2.16.0 software (National Institute of Health, Bethesda, MD, USA). Six replicate tissues were analyzed for each condition (n = 6), except for the vagina with fresh AA, where n = 5, and the skin and vagina with frozen AA, where n = 4 and n = 5, respectively.

### 2.12. Tissue-Scale Mechanical Properties

The mechanical properties of the constructs were assessed by uniaxial tensile testing using an ElectroPuls E1000 mechanical tester (Instron, Norwood, MA, USA) as previously described [40]. Bone-shaped biopsies were cut with a custom stainless-steel punch, with an inner width of 3 mm. Each extremity of the specimen was fixed between clamps and stretched at 0.2 mm/s until the tissue ruptured. Tissue thickness was measured using ImageJ software to analyze Masson’s Trichrome tissue slices, and the average tissue thickness was calculated based on at least five field averages per construct. Data was analyzed using Scilab (Dassault Systèmes, Vélizy-Villacoublay, France). Six replicate tissues were analyzed for each condition (n = 6), except for the vagina with fresh AA, where n = 5, and the skin and vagina with frozen AA, where n = 4 and n = 5, respectively.

### 2.13. Cell-Scale Mechanical Properties

The tissue samples were stored in a −20 °C freezer, and they were securely packed in a box containing dry ice for transportation to the atomic force microscopy (AFM) facility. To prepare a tissue specimen for force spectroscopy, the specimen was thawed at room temperature with 1X phosphate-buffered saline (PBS), then it was placed on a 50 mm × 75 mm microscope glass slide (Brain Research Laboratories, Waban, MA, USA). To ensure tissue hydration, several drops of 1X PBS were applied to the tissue surface. To preserve the PBS on the tissue surface, a hydrophobic circle was drawn using ImmEdge^®^ Hydrophobic Barrier PAP pen (Vector Laboratories, Inc., Newark, CA, USA) around the intended placement area prior to position the tissue on the glass slide.

The stiffness of the tissue samples was measured using MFP-3D Asylum atomic force microscopy (Asylum Research, Oxford Instruments, Santa Barbara, CA, USA), equipped with a silicon nitride cantilever with a nominal spring constant of 0.06 N/m and a 5 µm-diameter borosilicate glass spherical tip (Novascan Technologies, Inc, Boone, IA, USA). Upon mounting a cantilever on the AFM, its actual spring constant was measured in 1X PBS using the thermal tune method. For all conditions, a nominal force–distance cycle length of 10 µm, a PIEZO velocity of 3 µm/s, and a trigger force value of 7 nN were established. Each sample group contained 3 tissue specimens. To obtain a valid estimate of the tissue stiffness, 6 × 6 force maps with the scan area of 100 µm × 100 µm were acquired in at least 3 arbitrary regions of each tissue specimen (except for 1 single tissue specimen, on which two 6 × 6 and one 4 × 4 force maps of the same scan area were acquired). This led to the generation of stiffness heat maps with a 20 µm orthogonal distance between adjacent indentation points. The stiffness values were obtained by fitting the force curves to the Hertz contact model, assuming a tissue Poisson’s ratio of ν = 0.45. The fitting was performed up to an indentation depth of 625 ± 1 nm from the initial contact point. For the curve fitting analysis, only the curves demonstrating a stable and reliable tip–surface interaction were considered. The curves showing instability or poor fit to the model were excluded from further analysis.

### 2.14. Quantitative Polarized Light Microscopy

All constructs were fixed in a 3.7% neutral buffered formaldehyde solution and embedded in paraffin blocks. Tissue cross-sections of 5 µm were stained with picrosirius red and imaged using quantitative polarized light microscopy (QPOL), as described previously [43]. Briefly, an Axio Observer.Z1/7 microscope (Zeiss) was modified by adding a linear motorized polariser (Thorlabs, Newton, NJ, USA; max speed of 20 s^−1^) above the condenser and a circular polarizer in the imaging plane. Using monochromatic red light, an image sequence of 20o increments was acquired over 180o using an EC Plan-Neofluar 20x/0.50 Pol M27 objective and an Axiocam 305 monochromatic camera (Zeiss). From this sequence of images, the optical retardance signal was then measured and processed into a color-coded image using a custom python code. After applying a median filter, the retardance images were analysed using the OrientationJ plugin in FIJI to obtain the distribution of orientation curves. A Matlab code was then used to shift the curves, so the closest peak (main peak) was set at 0o, representing the horizontally laid collagen fibers and tissue orientation. The main peak prominence and the width at 1/2 prominence were also measured for each curve. Six replicate tissues were analyzed for each condition (n = 6), except for the vagina with fresh AA, where n = 5, and the skin and vagina with frozen AA, where n = 4 and n = 5, respectively.

### 2.15. Statistical Analyses

A one-way nested ANOVA, followed by post-hoc Tukey’s tests, was conducted (GraphPad Prism 8, GraphPad software, Boston, MA, USA). The probability level was considered significant at *p* < 0.05.

## 3. Results

### 3.1. Influences of Donor Origin and Supplements on Pro-Collagen 1α1 Secretion

**Figure 2:**
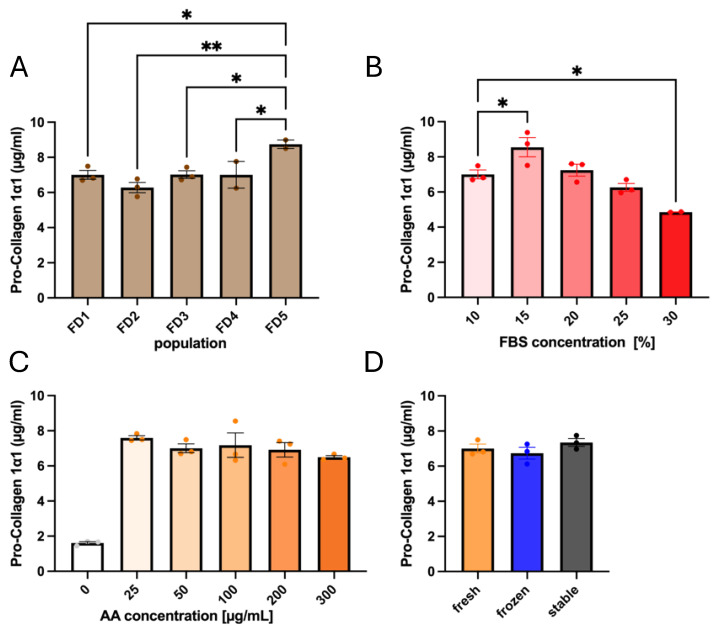
Quantification of pro-collagen 1α1 secretion by fibroblasts after 72 h. Evaluating 5 populations of dermal fibroblasts with 50 µg/mL AA (**A**). FD1 was used for the following: to assess the effects of varying concentrations of FBS (**B**) and AA (**C**), using 50 µg/mL of freshly prepared or frozen AA, or 2PAA (**D**). Error bars indicate SEM. * indicates 0.05 > *p* > 0.01, and ** indicates 0.01 > *p* > 0.005. Comparison between 0 µg/mL AA control and other conditions gave *p* < 0001. n = 3 for all groups, except FD4, FD5, and 30% FBS, where n = 2.

Considering that our primary fibroblasts were from different donors, we first investigated if there was any variation in collagen secretion between donors. Since collagen is secreted in its pro-collagen form, composed of two 1α1 and one 1α2 strands, and needs to be cleaved before they can assemble into fibrils, the amount of pro-collagen 1α1 in solution provides an effective readout of the collagen secretion. In this context, we evaluated the amount of pro-collagen 1α1 present in the media of cells in culture in the presence of AA (Figure 2A). Only the population FD5 showed a statistical difference compared to the 4 other populations, where collagen secretion was 28% higher than the average of the other 4 (8.74 ± 0.40 µg/mL for FD5 compared to 6.81 ± 0.29 µg/mL for the 4 other donors). This tells us that inter-population variability for collagen baseline secretion was limited when media were supplemented with AA. Moreover, the serum concentration had little impact on pro-collagen 1α1 secretion compared to the effect of AA (Figure 2B). Collagen secretion levels peaked at 15% FBS and decreased as serum concentration was further increased. Only a slight increase was seen for 15% FBS compared to 10% (8.55 ± 0.91 µg/mL and 7.00 ± 0.41 µg/mL, respectively).

AA is commonly used at 50 µg/mL, as it can become cytotoxic at higher concentrations [44]. Thus, to confirm that 50 µg/mL was an adequate concentration, we extended our analysis of collagen secretion to a higher concentration of AA. The evaluation showed no significant difference in pro-collagen 1α1 secretion between 25 and 300 µg/mL, indicating a saturation of the potential of AA for collagen production (Figure 2C). Therefore, the standard 50 µg/mL AA concentration was kept for the following steps. We then tested whether the AA storage condition impacted collagen secretion. Storage was evaluated by comparing the AA that had been freshly prepared or previously stored at −80 °C, as well as 2PAA, for an initial evaluation of collagen secretion (Figure 2D). There was no significant difference between the three groups, indicating that, contrary to current practices [45], AA does not have to be prepared fresh at each media change if stored correctly, and that 2PAA induces collagen secretion similar to that induced by AA.

### 3.2. Ascorbate Stability in Both Storage and Culture Conditions

**Figure 3:**
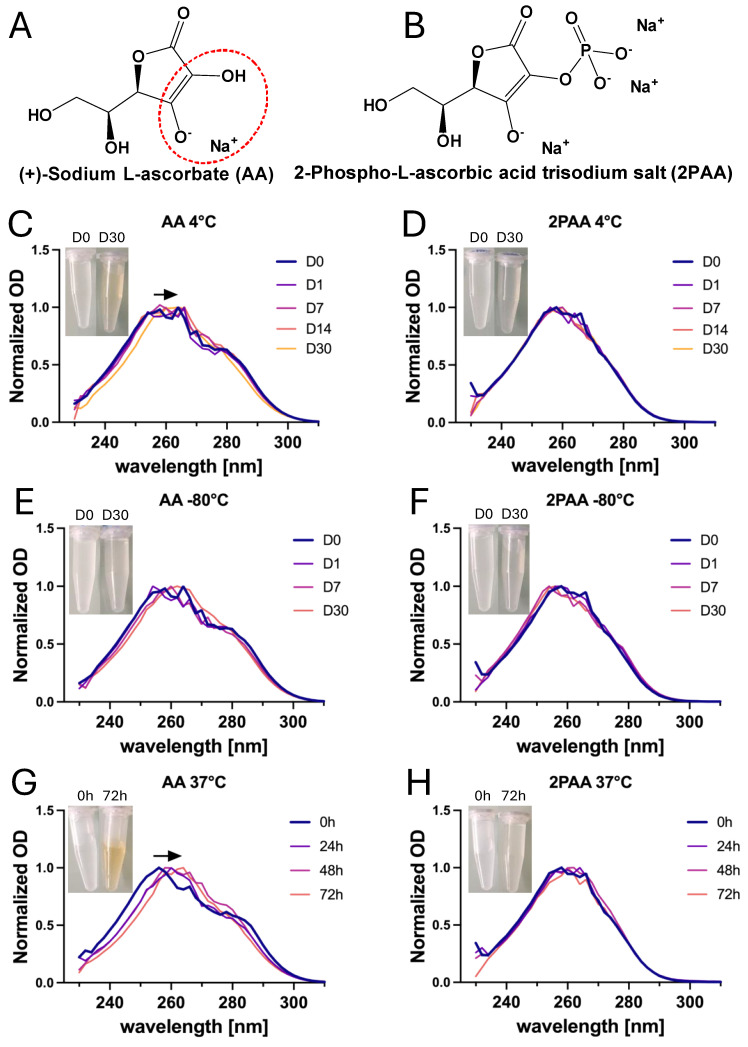
Stability evaluation of AA and 2PAA at different storage temperatures by UV–vis spectroscopy. Visual representation of AA (**A**) and 2PAA (**B**). The dashed Rred circle indicates a region susceptible to oxidation. Absorption spectra of AA and 2PAA solutions stored up to one month at either 4 °C (**C**,**D**) or −80 °C (**E**,**F**). Absorption spectra of AA and 2PAA solutions in cell-culture conditions (37 °C, 5% CO_2_ incubator, (**G**,**H**)). OD indicates optical density. Macroscopic aspects of the stock solutions (10 mg/mL) are illustrated in inserts. The arrows indicate a shift in the maximum absorption peak occurring during the degradation of the molecule.

The role of AA as a hydroxylase cofactor depends on its oxygen groups, and oxidation reduces its ability to upregulate collagen production [46]. Therefore, we assessed AA and 2PAA oxidation status by direct UV–vis spectrophotometry, which can be used to quantify both molecules in phenol-free culture media [47]. The stock solutions were diluted to 50 µg/mL in DMEM and analyzed under acquisition conditions (25 °C, pH 7.4). Both AA and 2PAA presented a maximum of absorption peak at around 260 nm. The stability of AA and 2PAA was evaluated for storage conditions (i.e., 4 °C or −80 °C) with the aim of facilitating routine changes of cell culture media (Figure 3C–F). The shift of the absorption maximum of AA between 2 weeks and 1 month of storage at 4 °C, as well as the yellowing of the solution, indicated that AA degrades over time in this condition. This trend was less clear when the AA solution was stored at −80 °C but it was still present, as the spectra did not overlap perfectly. For 2PAA, no change in the absorption spectra or in the color of the solution was observed over 1 month of storage at either 4 °C or at −80 °C. These results indicate that 2PAA can be prepared in advance for at least one month and stored at 4 °C for cell culture media changes. In contrast, it appears AA can be stored properly for at least 2 weeks at 4 °C.

Next, we explored AA versus 2PAA stability under standard cell-culture conditions. For this experiment, we stored cell culture media supplemented with a working dilution of AA or 2PAA (50 µg/mL in DMEM), at 37 °C in a humidified 5% CO_2_ incubator for up to 72 h, the longest time interval between cell culture medium changes during weekends (Figure 3G,H). The AA spectra presented a shift in the maximum of absorbance toward higher wavelength over time, while 2PAA absorption spectra were superimposed at all timepoints. The degradation of AA was macroscopically observed through the yellowing of cell culture medium over time. Thus, it appears that 2PAA is highly stable in cell-culture conditions, in contrast to AA.

### 3.3. Impact of AA and 2PAA on Engineered Stroma Composition

**Figure 4:**
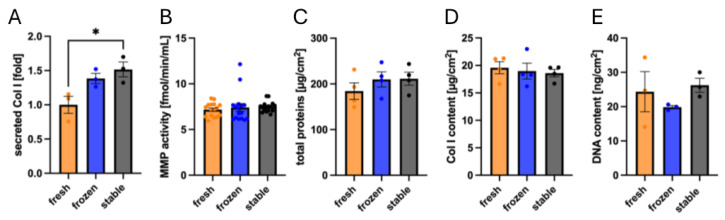
Biochemical evaluation of engineered dermal stroma produced with fresh or frozen AA or 2PAA. Western blot of Col I secreted in the media (n = 3) (**A**). Quantification of MMP activity in the media (N = 4, n = 4) (**B**). Total non-collagenous proteins in the engineered stroma quantified by Fast Green (n = 4) (**C**). Total deposited Col I quantified using Sirius Red (n = 4) (**D**). DNA content in the engineered stroma quantified using the PicoGreen assay (n = 3) (**E**). * indicates 0.05 > *p* > 0.01.

Considering our results on the AA and 2PAA stability under cell-culture conditions, we proceeded to evaluate the dermal stroma composition to elucidate any molecular differences between tissues produced in the three ascorbate conditions. Since collagen is secreted by fibroblasts into the media, where it matures before being deposited to form the ECM [48], the non-deposited type 1 collagen in the supernatant was first quantified by Western blot (Figure 4A), revealing a 52% increase in secretion for tissues cultured in 2PAA compared to freshly prepared AA. This is probably due to the prolonged bioavailability of 2PAA in the culture media compared to the rapidly degrading AA. Interestingly, the tissues made with frozen AA did not show a significant difference between either fresh AA or 2PAA groups.

MMPs are critical ECM-remodeling enzymes, and when the stroma sheets are stacked, MMP activity is supposed to increase to ensure proper sheet fusion [49]. Thus, we proceeded to assess MMP-specific activity for the different ascorbate conditions and the quantification revealed no difference (Figure 4B), indicating that tissue remodelling is at a similar level in the 3 conditions.

Finally, we performed a simultaneous quantification of total collagen and non-collagenous protein content in the engineered stroma using Sirius Red–Fast green, which showed a similar non-collagenous protein content in all three conditions (Figure 4C). Moreover, collagen quantification within the tissue showed similar levels for the three conditions (Figure 4D), indicating that despite an increase in collagen secretion in the supernatant, a similar quantity of collagen was deposited. A PicoGreen assay allowed for the quantification of DNA in the tissues and displayed no significant differences (Figure 4E).

Together, these results indicate that, despite the increased collagen synthesis in tissues produced with 2PAA, tissue composition remains similar for tissues produced with either fresh or frozen AA or 2PAA in terms of cell content, as well as collagen and other protein content, and remodeling activity.

### 3.4. Impact of AA and 2PAA on the Physical Properties of Engineered Stroma 

**Figure 5:**
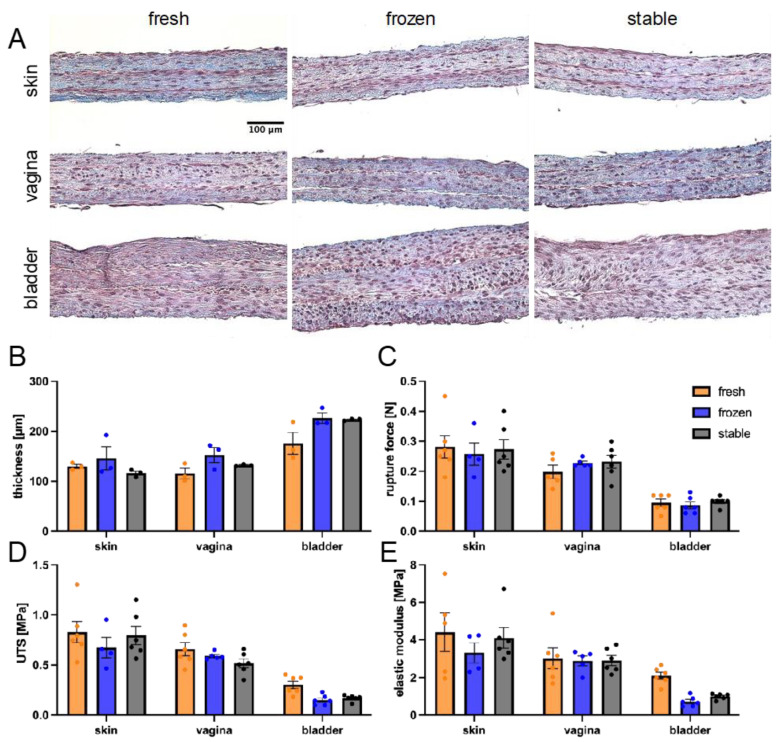
Mechanical properties of stroma produced with fresh or frozen AA or 2PAA. Masson’s trichomes of skin, vagina, and bladder stroma engineered using fresh or frozen AA, or 2PAA (**A**). Average thicknesses of engineered stroma (**B**). Rupture force of tissues evaluated using a bone-shaped punch (**C**). Ultimate tensile strength (UTS, (**D**)) and elastic modulus (**E**) of engineered tissues. n = 6 for each condition, except for the vagina with fresh AA, where n = 5, and skin and vagina with frozen AA, where n = 4 and n = 5, respectively.

Besides overall protein content, the mechanical properties of tissues also play a critical role in maintaining physiological functions [50]. Tissues were produced with fibroblasts of dermal, vaginal, or bladder origin under each respective ascorbate condition to evaluate differences in histoarchitecture and mechanical properties. One population was representative for each organ. Tissues were stained with Masson’s Trichrome (Figure 5A), and tissue thickness was measured for three replicas (Figure 5B). Between organs, there is a natural variation in self-assembled tissue thickness. The bladder stroma was thicker than both the skin and vaginal stroma. For each organ, the ascorbate conditions produced tissues with a similar thickness. Interestingly, tissues made with 2PAA showed less variability within a given tissue than those made with AA, with a coefficient of variation of 5.7 ± 2.7% compared to 14.7 ± 6.7%, respectively, and a *p*-value of 0.065, n = 3. This indicates that the constructs are more homogenous. It was also noted that the fresh AA condition showed poorer fusion compared to the frozen and stable ascorbate.

Mechanical properties were evaluated for the 6 reconstructed tissues by uniaxial traction (Figure 5C–E). A large variability was observed, potentially due to the relatively short fusion time and tissue delamination, which may have induced a bias. Significant differences were observed between organ types: skin and vaginal stroma had similar properties, while bladder stroma were weaker, despite being thicker than both skin and vaginal tissues. For both elastic modulus and ultimate tensile strength, there was no significant difference observed between ascorbate conditions for any of the three organs.

**Figure 6:**
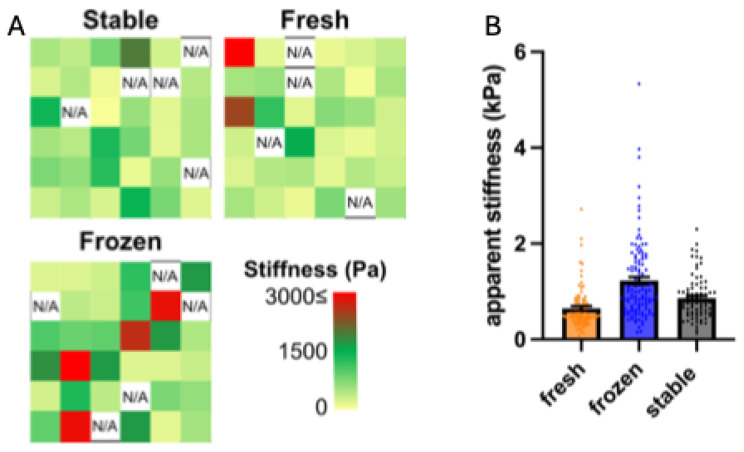
Cell-scale mechanical properties of engineered stroma evaluated using atomic force microscopy. Representative heat maps of stiffness measurements within a given tissue (**A**). Stiffness of stroma produced with fresh or frozen AA or 2PAA (**B**). N/A indicates unattainable values. n = 3.

The mechanical properties measured with uniaxial tensile testing are not necessarily representative of cell-scale mechanical properties of a tissue due to the innate heterogeneity of the ECM [51,52]. In order to evaluate the micron-scale mechanical properties, measurements were conducted. AFM lends itself as an ideal method for this evaluation. By evaluating stroma made with FD, it was noticed that each tissue presented stiffness heterogeneity throughout its surface, ranging from ~200 to 3000 Pa (Figure 6A). Although the three ascorbate conditions gave similar average stiffness measurements (Figure 6B), it was noticed that the stiffness of tissues made with AA, particularly the frozen AA, were more heterogeneous compared to those made with 2PAA.

### 3.5. Impact of AA and 2PAA on Collagen Architecture of Engineered Stroma

**Figure 7:**
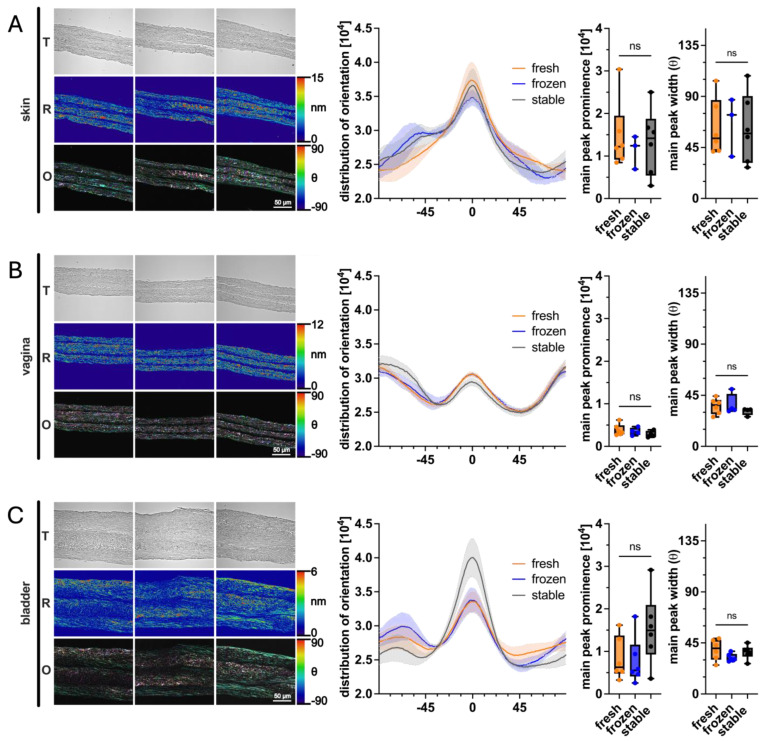
Collagen alignment within engineered stroma evaluated with quantitative polarization microscopy. Evaluation of stroma produced with dermal (**A**), vaginal (**B**), or bladder (**C**) fibroblasts with fresh or frozen AA or 2PAA. The left panel represents transmittance (T) images, and retardance (R) images, which are then converted to orientation (O) measurements. Graphs represent from left to right, the distribution of collagen orientation, peak prominence, and peak width. n = 6 for each condition, except for the vagina with fresh AA, where n = 5, and the skin and vagina with frozen AA, where n = 4 and n = 5, respectively. ns indicates no significant difference.

Collagen fibril organization is important for a tissue’s structural properties and plays a role in cell-signaling, which is crucial for tissue-specific behavior [53]. For this experiment, QPOL was employed to study the impact of fresh/frozen AA or 2PAA on the orientation and organization of the collagen fibrils deposited in three different types of reconstructed tissues (Figure 7). The results show that the use of stable 2PAA, compared with fresh or frozen AA, showed no significant differences in the main peak width and prominence of the collagen fiber orientation distribution. However, in the bladder tissues, a slight trend was observed, where the 2PAA condition tended to lay more horizontally aligned collagen fibers, as seen in the higher main peak prominence.

Taken together, these results highlight that the use of frozen AA aliquots does not impact engineered tissue production and, importantly, that engineered tissues produced with 2PAA are as effective and more homogeneous than those made with AA, making 2PAA an effective replacement for AA.

## 4. Discussion

This study demonstrated that 2PAA is a viable substitute for AA for inducing ECM production by fibroblasts for the use in tissue engineering applications. The use of 2PAA resulted in tissues that were structurally and compositionally comparable to those produced with freshly prepared or frozen AA, with no significant differences observed in key biochemical and mechanical parameters. A control condition without ascorbate was not included, as the self-assembly method depends on ascorbate-induced collagen secretion. Importantly, the tissues made with 2PAA exhibited more uniformity and reduced variability both within the tissue and between replicates. Additionally, 2PAA’s increased stability under cell-culture conditions offers practical advantages, such as decreased handling time and reduced material use. Since stability was the only controlled difference between conditions, it is likely the main factor contributing to the observed consistency. These findings highlight 2PAA as a promising alternative to AA, providing potential cost reductions and operational efficiency improvements in tissue engineering applications. Importantly, the reduced variability between batches and increased process robustness address major barriers in clinical translation, such as standardization and reproducibility, which are critical for Good Manufacturing Practice (GMP) compliance and the production of clinical-grade tissue.

While this research is particularly relevant for researchers focused on the self-assembly method [33], the findings extend far beyond. Fibroblasts, and fibroblast-like cells, are widely used in tissue engineering applications, including scaffold-based approaches, where the production of ECM is critical [54]. Since AA is commonly added to stimulate ECM production, our findings indicate that 2PAA can serve as an effective, stable alternative for any researcher looking to enhance fibroblast-mediated ECM synthesis. The broader implications of these results are relevant to anyone aiming to produce strong and functional ECM in various tissue engineering contexts, potentially accelerating developments across a wider spectrum of regenerative medicine applications. As with most products in regenerative medicine and tissue engineering, standardization and scale-up have been a barrier to large-scale clinical translation, with only a few clinical trials having been performed to date, despite promising results both in vitro and in vivo in creating functional replacement organs [55]. The ability to use a stable alternative such as 2PAA simplifies workflows and reduces the frequency of quality control interventions, which can facilitate regulatory approval and the implementation of scalable manufacturing pipelines. Small or miniaturized tissue engineering constructs such as tissues on a chip or multi-well sized constructs have been used for research models of a large array of tissues to predict healthy and pathological tissue responses to stimuli [56,57]. By optimizing the production protocol and reducing costs, these models would be more widely available to be used in vitro. Given the ethical dilemma of using animal models, as well as the translation issues widely encountered when drawing evidence from nonhuman models, there is a large demand for accessible in vitro organ models [58]. The cosmetics industry in particular is in need of alternative testing methods considering the 2013 ban on animal testing in the EU [59], and with other countries following suit. Tissue engineering provides advanced tissue models with native characteristics that are tunable for various organs. A cell-produced, tissue-specific ECM provides an advantage in achieving a native-like tissue in vitro, capable of provide clinically relevant results [54].

As AA preparation is a significant contributor to high production costs, a stable alternative was sought out for the molecule, which would reduce costs and optimize the tissue production method without impacting the properties of the final construct. Currently, AA is prepared fresh at each media change. This requires weighing the molecule, followed by suspension and sterile filtering, then measuring the correct volume to add to the culture media. 2PAA, known for its stability, was identified as a potential replacement. Using 2PAA allows for large-batch media preparation, reducing technician time and product waste. The assays confirmed 2PAA’s stability, whereas AA degraded rapidly under culture conditions. However, AA remained stable at 4 °C for up to 2 weeks, allowing biweekly preparation. AA could also be stored at −80 °C for at least a month, eliminating the need for frequent sterile filtering. The instability of AA in culture conditions, however, raises questions as to the molecule availability for inducing collagen synthesis if the molecule is mostly degraded before the media are refreshed. Hata and Senoo performed a similar experiment in 1989, where AA and 2PAA were measured when stored at 37 °C or 5 °C [39]. They observed similar results, where 50% of AA was degraded after 1 day, and nearly all was lost after 3 days at 37 °C. At 5 °C, they observed a 50% reduction after 7 days and the complete degradation of AA after 30 days. They did not observe significant degradation of 2PAA at any of the measured timepoints. Our conclusions align in that AA rapidly degrades at 37 °C but may be stored in the fridge for several days, and that 2PAA does not easily degrade in solution, which makes 2PAA very attractive for tissue engineering applications.

As skin in vitro models are increasingly important to both academia and industry [60], the biochemical analysis focussed on dermal fibroblasts. Despite measurable AA degradation under culture condition, a sufficient concentration of pro-collagen is most likely secreted by the fibroblasts over the course of 72 h to enable the formation of a stroma. These results align with prior studies, such as those by Hata, which observed little difference in collagen synthesis across a wide range of AA concentrations (20–200 µg/mL) [39,61]. Similarly, we found that 2PAA stimulated pro-collagen secretion at levels comparable to AA, corroborating Hata and Senoo’s findings [39]. This suggests that while the AA concentration can fluctuate within a certain range, its impact on collagen production remains consistent, offering flexibility in tissue engineering protocols. This may be due to the saturation of produced collagen in the media. Foroughi et al. revealed that collagen secretion can be increased by exposing fibroblasts to a pulse-bioreactor. However, collagen deposition was not different from static conditions [62]. Moreover, the stability of 2PAA could minimize the degradation and variability observed in AA-treated cultures, providing more predictable outcomes in clinical applications. In the context of scaffold-based tissue engineering and other fibroblast-driven ECM production methods, this stability and efficiency make 2PAA a highly attractive alternative. In fact, we observed a similar trend in both the bladder- and vagina-engineered stroma, where 2PAA did not result in increased collagen deposition. Future studies could explore the potential of 2PAA in enhancing ECM deposition in various cell types, including chondrocytes and osteoblasts, as well as in three-dimensional culture systems, where matrix composition and organization are vital to mechanical integrity and function [63].

Tissue structure and mechanical properties are paramount in self-assembly tissue engineering, as there is no scaffold to support the tissue if it were to be surgically grafted. The mechanical properties of tissues are most often measured via macroscopic tests, such as uniaxial tension [64]. While these tests do not take in to account the unique ECM structure and physiological mechanical strain [51], they provide a modulus that is relevant for surgical manipulation [65,66]. Such data are crucial when considering the application of engineered tissues as grafts, where surgical handling and implantation require a predictable mechanical response. Standardizing these properties is essential for clinical use. As tissue composition was shown to be similar across all conditions, tissue thickness was likewise similar across all conditions, as were the mechanical properties. Interestingly, the bladder tissues were weaker than the skin and vaginal tissues, despite being thicker. This probably originates from a different ECM composition, and it would be interesting to investigate further. Of note, our tissues were composed of three stacked stromal sheets to increase thickness and thus mechanical resistance. However, improper fusion between sheets was noticed in the AA condition, which could explain the spread between the measured mechanical properties of those tissues. It is known that MMPs are released following tissue stacking, and the sheets are remodeled to fuse together [49]. Of note, fully functional bilayered tissues are obtained by seeding epithelial cells on the engineered stroma [33]. To our knowledge, stromal delamination after a subsequent month of co-culture with epithelial cells has not been reported as an issue despite the use of AA, suggesting this issue can either be medicated or has not been properly documented. Differences in stromal sheet fusion may indicate something beyond soluble MMP activity that was measured, as it was shown to be stable. MMP14, for example, is membrane-bound [67] and would not have been accounted for in supernatant evaluation. In addition, we observed differences in terms of mechanical heterogeneities at the microscale level. Therefore, the role of other actors of collagen remodeling could be investigated, such as the lysyl oxidase (LOX) enzymes, which are responsible for the crosslinking of ECM proteins. For instance, an increase in LOX activity results in the stiffening of the ECM [68]. The stimulation of LOX in engineered tissues was thought to be a way to increase tissue strength. However, LOX upregulation will induce the stiffening of the tissues, and once it reaches pathological levels, it may negatively impact epithelial cells exposed to such a stiff matrix [69]. Other characteristics of the tissues appeared unchanged, such as MMP expression, total non-collagenous proteins, and DNA content. Overall, our results highlight that tissues produced with 2PAA have nearly identical mechanical properties to those produced with AA, whether freshly prepared or stored at −80 °C.

Markedly different values were observed when comparing the macroscopic and microscopic mechanical properties. For instance, the cells experience a stiffness of around 2 kPa in dermal constructs, whereas the tissue has a modulus of around 4 MPa. Such a difference is expected from the viscoelastic characteristic of soft tissues [70]. It is important to know both values, as engineered tissues are used both as graft sources and in preclinical models. The macroscopic modulus is most important in a clinical context, where the tissue needs to be surgically manipulated. Once grafted, the tissue should reorganize to conform to native tissue properties. For preclinical models, the cell-scale environment takes precedence, as cellular behavior is strongly influenced by the mechanical properties of the ECM [71]. In such a context, the increased uniformity achieved with 2PAA might be an advantage.

Collagen conformation is a subject that is often ignored in self-assembly tissue engineering, as much attention is given to the epithelium rather than the stroma. In native tissues, collagen is oriented along different axes to optimize strength according to the requirements of the tissue, as aligned collagen is more resistant to tensile force than scattered fibers. For example, Langer’s lines describe the orientation of collagen fibers in the dermis and are generally parallel to the underlying muscle fibers [72]. Tendons, for example, are mostly composed of precisely aligned and dense collagen fibers, as their principal role is to bear a significant tensile load along their principal axis. Self-assembled tissues are thought to deposit collagen fibers in a random orientation along the length of the tissue; however, studies have shown that tensile load-inducing bioreactors are able to align collagen fibers and increase the strength of a tissue along the axis of tension [73]. The alignment of deposited collagen fibers was evaluated by quantifying the polarization of Sirius Red staining in collagen fibers. This technique allows for the quantitative analysis of the ECM structure within a tissue section. There were some interesting trends that appeared between tissue sources but not between ascorbate conditions, meaning that 2PAA did not affect collagen deposition orientation. Collagen fibers were mostly aligned along the length of the tissue, as expected; however, the amount of non-longitudinal collagen fibers was significant, especially for bladder tissues. In vivo evaluation of stromal maturation would be interesting to observe how collagen alignment may evolve according to tissue type and graft site.

## 5. Conclusions

Overall, few differences were found between AA and 2PAA in tissue production. Despite potentially higher collagen synthesis, tissue composition was not significantly different in any of the evaluations performed. Notably, the tissues produced with 2PAA tended to be more uniform, with less variability, which is important for reproducibility in tissue engineering. The tissues produced with 2PAA tended to be more uniform, with less variability. By using 2PAA in the production of self-assembled tissues, both material usage, including costly filters, and technician time required for media preparation over long culture periods can be reduced. By accounting for the savings achieved, the higher price of 2PAA compared to AA is justified and ends up reducing ascorbate related costs by 8.3-fold. It was also observed that if AA is stored at 4 °C, it is stable for around 2 weeks, indicating that simple changes could be made to protocols to gain in efficiency and help make this technology accessible. 2PAA is already used in the cosmetics industry thanks to its stability, and its use in tissue engineering could help improve clinical translation and scale-up by allowing the use of automated cell culture machines, which would dramatically reduce production costs in the long term by limiting costly human labor in maintaining tissues in culture. While this study focused on fibroblast-based self-assembled stromal tissues, 2PAA should be evaluated in other models, such as organoids or biomaterial-based constructs, to test its efficacy more broadly. It may also be valuable to assess its effects on additional mesenchymal populations, such as adipose-derived stem cells (ASCs) and chondrocytes, where ECM production is crucial for some models. Due to the inherent variability in primary cells from different donors, some potentially meaningful differences could not be statistically confirmed in this study. This limitation underscores the need for future research, with larger sample sizes and more diverse cell sources to better elucidate the effects of 2PAA across different biological contexts and strengthen the generalizability of our findings.

## Data Availability

Data are available upon request to the corresponding authors.
